# Genotype by environment interaction and breeding for robustness in livestock

**DOI:** 10.3389/fgene.2015.00310

**Published:** 2015-10-20

**Authors:** Wendy M. Rauw, Luis Gomez-Raya

**Affiliations:** Departamento de Mejora Genética Animal, Instituto Nacional de Investigación y Tecnología Agraria y Alimentaria, Madrid, Spain

**Keywords:** livestock production, animal breeding, genetic selection, robustness, reaction norms, phenotypic plasticity, canalization

## Abstract

The increasing size of the human population is projected to result in an increase in meat consumption. However, at the same time, the dominant position of meat as the center of meals is on the decline. Modern objections to the consumption of meat include public concerns with animal welfare in livestock production systems. Animal breeding practices have become part of the debate since it became recognized that animals in a population that have been selected for high production efficiency are more at risk for behavioral, physiological and immunological problems. As a solution, animal breeding practices need to include selection for robustness traits, which can be implemented through the use of reaction norms analysis, or though the direct inclusion of robustness traits in the breeding objective and in the selection index. This review gives an overview of genotype × environment interactions (the influence of the environment, reaction norms, phenotypic plasticity, canalization, and genetic homeostasis), reaction norms analysis in livestock production, options for selection for increased levels of production and against environmental sensitivity, and direct inclusion of robustness traits in the selection index. Ethical considerations of breeding for improved animal welfare are discussed. The discussion on animal breeding practices has been initiated and is very alive today. This positive trend is part of the sustainable food production movement that aims at feeding 9.15 billion people not just in the near future but also beyond.

## Animal Breeding and Animal Welfare

Although an increase in overall meat consumption is expected in the coming decades resulting from the ever growing human population, the dominant position of meat as the center of meals is on the decline. This is motivated by religious, health, moral, and environmental considerations. Rauw (2015) reviewed the history of ethics of animal use and consumption from Pythagoras to Bentham (c 500 BC to the end of the 18th century), which describes the origins of health and moral objections to the consumption of meat. Of a much more modern origin are environmental considerations, and public concerns with animal welfare in livestock production systems; the latter particularly came about in response to the publication of Harrison’s (1964) book “Animal Machines: the New Factory Farming Industry”. Rapid turnover, high-density stocking, and a high degree of mechanization resulted in a public awareness of the results of intensification of livestock production practices and “factory farming” in the 60s and resulted in an increasing number of philosophical writings on animal rights from the 70s on (Singer, 2005; Stamp Dawkins, 2013). Factory farming is characterized by overcrowding, restricted movement, unnatural diets and unanesthetized surgical procedures resulting in physical pain and necessarily in reduced animal welfare (Frank, 1979). Frank (1979) suggested that intensive farming differs from factory farming in that it involves increasing productivity through better management and breeding techniques but without necessarily involving crowding and thus significantly altering the pattern of life the animal leads. However, this situation no longer applies since it became recognized that animals in a population that have been selected for high production efficiency are more at risk for behavioral, physiological and immunological problems (Rauw et al., 1998). Examples are most pronounced in populations that are selected for narrow yield goals at high intensity of selection, such as broiler chickens selected for increased body weight at a certain age (Rauw et al., 1998; Rauw, 2009). As Oltenacu and Algers (2005) write regarding dairy cattle: “[Improved production efficiency] should optimize the use of resources, increase farm profit, and reduce cost for consumers. In many European countries, yield per cow has more than doubled in the last 40 years. The dramatic increase in yield per cow is due to rapid progress in genetics, nutrition and management,” however, due to the resulting fertility problems, increasing incidence of health problems, and declining longevity in modern dairy cows, “genetic selection for increased milk yield increasingly is viewed as increasing profit at the expense of reducing animal welfare.”

As a result, animal breeding practices have become part of the debate that deals with issues of animal welfare and animal production ethics and at a wider scope with sustainable agriculture and livestock production. Frank’s (1979) definition of intensive farming practices which do not negatively affect the pattern of life of the animals involved is now newly captured under the banner of “sustainable intensification” of livestock production, i.e., improving productive output while maintaining animal health and welfare (Gamborg and Sandøe, 2005; Charles et al., 2014). The Farm Animal Welfare Council has emphasized welfare concerns in relation to animal breeding strategies since 1992 in their reports (FAWC, 2004, 2012; MacArthur Clark et al., 2006). For example, the 1992 report on the welfare of broiler chickens reads: “Genetic selection has the potential for positive as well as negative effects on welfare. However, the selection of stock for liveweight gain and food conversion efficiency in preference to, and to the detriment of, factors necessary for the welfare of the birds should be discouraged” (FAWC, 2004). The 1998 Council Directive 98/58/EC concerning the protection of animals kept for farming purposes reads: “Natural or artificial breeding or breeding procedures which cause or are likely to cause suffering or injury to any of the animals concerned must not be practiced” (EUR-Lex, 2015). In 2000, the Sustainable European Farm Animal Breeding and Reproduction project was initiated by the Farm Animal Industrial Platform (currently the European Forum of Farm Animal Breeders); one of the aims was an agreement by breeding organizations to develop Codes of Practice (MacArthur Clark et al., 2006; Neeteson-van Nieuwenhoven et al., 2006). The main objectives of the resulting Code-EFABAR launched in 2006, a voluntary “Code of Good Practice,” are to be the standard instrument for defining and maintaining good practices for farm animal breeding, and to create transparency for society (Code-EFABAR, 2006). As MacArthur Clark et al. (2006) conclude, failure to address the issues arising from bad breeding practices presents a significant risk to Governments, to the livestock industry, and to animal welfare.

## How *should* we Breed?

Animal production is basically an input-output system to which the first law of thermodynamics, or the law of conservation of energy, applies, in the same way as it does for any other energetic system: energy cannot be created nor destroyed, but can only be changed from one form to another. Energy in output (production, losses) requires an equal amount of energy input (eventually this comes down to food intake). In other words: an animal from a population genetically selected for increased production will only be able to realize this potential in an environment in which resources are adequately supplied (Beilharz et al., 1993; Rauw, 2009). However, while this holds even intuitively, in practice, livestock animals are often genetically selected for *increased* levels of production (output) at the same time that they are selected for *decreased* levels of energetic input (improved feed efficiency, decreased levels of fatness; Rauw, 2012). A clear example of selection practices that have resulted in a mismatch between input and output is the voluntary feed intake capacity of young sows which has been reduced as a consequence of selection for high lean growth, resulting in animals that are constrained by limited body reserves and/or limited feed intake capacity at the time of lactation when they have to support a genetically increased litter size and growth rate. As Knap (2005) writes regarding pig production: “Increasing genetic potential requires advances in animal nutrition and animal management to support its expression, but these advances have often been poorly addressed or overlooked.” This results in the inability to maintain a successful balance of biological needs and consequently, inadvertently, in animals that are less robust, showing undesirable side effects of genetically improved levels of production (Siegel and Dunnington, 1997; Rauw et al., 1998; Knap, 2005).

In addition, livestock animals are required to perform in a wide variety of environmental conditions, regarding climate, housing facilities, social environment, disease pressure, and differences in feed quality and composition (Knap and Wang, 2006; Star et al., 2008; Mormède et al., 2011). The farm animal of the future is thus described as robust, adapted, and healthy (Mormède et al., 2011), i.e., having “the ability to combine a high production potential (growing or reproductive) with resilience to stressors, allowing for unproblematic expression of a high production potential in a wide variety of environmental conditions” (Knap, 2005). After Knap (2005), the literature on selection for robustness traits has increased considerably, becoming a rapidly developing key area in farm animal breeding (Knap, 2009). Knap (2009) indicates that there are two options for breeding for animal robustness, which can be implemented simultaneously in an evaluation system for performance-relevant robustness: through the use of reaction norms analysis by estimating breeding values for the environmental sensitivity of the genetic potential for production performance (indirect approach), or through the inclusion of directly measurable robustness traits in the breeding objective and in the selection index (direct approach).

This review presents a historic overview of gene by environment interactions (including the concepts of reaction norms, phenotypic plasticity, canalization, and genetic homeostasis), the applicability of reaction norms analysis in livestock production, and the feasibility of selecting for the different reaction norm parameters (the level vs the slope). The review ends with a discussion of the feasibility of directly including robustness traits in the breeding objective and selection index, a discussion of the ethical consideration of selection for robustness, and with a short synthesis of all the material discussed in this paper.

## Genotype × Environment Interaction: A Historic Overview

### The Influence of the Environment

The influence of the environment on the phenotype and on evolution was of course most famously recognized by Jean Baptiste de Lamarck in his book “Philosophie Zoologique” in his chapter (translated) “Of the influence of the environment on the activities and habits of animals, and the influence of the activities and habits of these living bodies in modifying their organisation and structure” published in 1809. Indeed his statement that “the environment affects the shape and organisation of animals, that is to say that when the environment becomes very different, it produces in course of time corresponding modifications in the shape and organisation of animals (…) [because] great alterations in the environment of animals lead to great alterations in their needs” has become a “truth, which, once recognized, cannot be disputed” (Lamarck, 1914). He thus recognized the continuous dynamic geological, climate, and geographic changes in the environment as opposed to a static world, and in order to adjust to these changes, organisms had to evolve (Mayr, 1972). According to Lamarck, because “nature is forced to submit her works to the influence of their environment, (…) this environment everywhere produces variations in them” (Lamarck in Shaner, 1927). Resulting from this, “Nature has produced all the species of animals in succession, beginning with the most imperfect or simplest, and ending her work with the most perfect, so as to create a gradually increasing complexity in their organisation, (…) [forming] a branching series, irregularly graded and free from discontinuity, or at least once free from it…” (Lamarck in Shaner, 1927). As Shaner (1927) notes, it was Lamarck who first thought of the animal kingdom as a great family tree, initiating the modern theory of evolution. However, to his disfavor, Lamarck is mostly known for his concept of inheritance of acquired characteristics formulated in his second law: “All the acquisitions or losses wrought by nature on individuals, through the influence of the environment in which their race has long been placed (…) are preserved by reproduction to the new individuals which arise” (Lamarck, 1914). This was similar to that proposed by Erasmus Darwin in his work “Zoonomia” published earlier in 1794: “[F]rom the first rudiment, or primordium, to the termination of their lives, all animals undergo perpetual transformations; which are in part produced by their own exertions in consequence of their desires and aversions, of their pleasures and pains, or of irritations, or of associations; and many of these acquired forms, or propensities, are transmitted to their posterity” (Darwin in Harrison, 1971). Darwin and Lamarck had failed to distinguish between the influence of the environment on individual animals (resulting in non-heritable modifications) vs. the influence of the environment on animal populations (resulting in evolution).

It was Erasmus’ grandchild Charles who successfully challenged the inheritance of acquired characters in individuals when he recognized the influence of the environment on evolution of animal populations, resulting from natural selection in the struggle for existence. But as to how variations were produced on which natural selection could act, he wrote: “I have hitherto sometimes spoken as if the variations—so common and multiform in organic beings under domestication, and in a lesser degree in those in a state of nature—had been due to chance. This, of course, is a wholly incorrect expression, but it serves to acknowledge plainly our ignorance of the cause of each particular variation” (Darwin, 1869). In an aim at answering this question of the origin of variation, he developed the hypothesis of pangenesis based on modifications and amplifications of earlier existing theories. Each unit of living tissue continually produced minute particles or “gemmules” at each stage of its development which would multiply and develop themselves into new cells and which were transmitted from parents to offspring via the reproductive organs (Geisen, 1969). This idea is similar to that proposed far back in antiquity by Hippocrates: “For the seed comes from all parts of the body, healthy seed from healthy parts, diseased seed from diseased parts” (Hippocrates in: Zirkle, 1946). However, not different from Lamarck, it was still a naïve conception of transmission of personal qualities as the heritable elements to the progeny.

### Reaction Norms and Phenotypic Plasticity

This approach to heredity was very different from the first controversial but accurate model by Mendel, first published in 1865 but not seriously considered until 1900, introducing “elements” of inheritance. These elements were later coined “genes” by Johannsen in 1909 and recognized as a segment of a chromosome after the discovery of the structure of DNA by Watson and Crick in 1953 (Portin, 2002). The discovery of Mendelian inheritance resulted in a temporary popularity of discontinuous “saltations” by mutations as the primary mechanism of evolutionary change as opposed to Darwin’s concept of evolution through natural selection acting on small continuous variations (Sarkar, 1999). Woltereck (1909), in order to prove Darwin right, studied phenotypic variation of continuous traits in morphologically distinct pure-line strains of Daphnia species subjected to variations in environmental factors. Plotting the response curves of the phenotypes (relative head height) of the different strains to the environmental variation (nutrient level) showed that the resulting *reaktionsnorm* (reaction norm, or standard pattern of the response curve) was different in the different strains (Woltereck, 1909; Sarkar, 1999). In his understanding, the genotype of an animal was synonymous to the shape of this curve, i.e., the reaction norm, and thus constituted the unit that was inherited, resulting in hereditary change. Johannsen, who had proposed the term “genotype” as the “sum total of all the “genes” in a gamete or in a zygote” agreed that “[t]he very appropriate German term “Reaktionsnorm” used by Woltereck is, as may be seen, nearly synonymous with “genotype,” in so far as the “Reaktionsnorm” is the sum total of the potentialities of the zygotes in question. (…) [It] emphasizes the diversity and still the unity in the behaviour of the individual organism; certainly, the particular organism is a whole, and its multiple varying reactions are determined by its “genotype” interfering with the totality of all incident factors, may it be external or internal. Thence the notion “Reaktionsnorm” is fully compatible with the genotype-conception” (Johannsen, 1911). However, he did contest that Woltereck’s observations disproved evolutionary saltations since he held that continuous transitions exhibited by phenotypes, as expressed in the reaction norm, result from discontinuous saltations in the genotype, i.e., through mutations.

Three years later, Nilsson-Ehle, discussed the “acclimatization or adjustment” to the climate by plants, i.e., “the plant’s ability to change their characteristics in one way or another such that it thrives in a new environment” (Nilsson-Ehle, 1914, quote translated from Swedish). Referring to a particular example of a 10-year study by Bonnier (1894), who described the adaptation of individual plants of the same genotype (cuttings of the same seedlings) to the climate at different altitudes in the Alpes and the Pyrenees with respect to their size, color, and shape, he concluded (translated from Swedish): “Summarizing all experience in this area, then you can also say that the climate’s influence can hardly be explained in a purely causal-mechanical way. One has to, as (…) even Johanssen explicitly holds, count with the organisms’ ability of self-adjustment or self-regulation, the appropriate reaction norm. This plasticity, depending on various external conditions, is in fact neither easier nor more difficult to interpret then the organism’s appropriate characteristics at all.” Nilsson-Ehle is by many recognized as being the first scientist to use the word “plasticity” (“plasticitet,” Nilsson-Ehle, 1914, p. 549) to describe the effect of the environment on the phenotype of an organism (Fuller, 2003), however, it was Bonnier himself who proposed it (“plasticité”) some 10 years earlier based on his own work that Nilsson-Ehle had referred to (translated from French): “The influence of the climate of the Alpine region is not only visible in the modification of the diverse exterior characteristics; it also has a profound effects on the development and the nature of the different tissues of the organism, each affected to a more or lesser extent. (…) Among the plants that support the climate change, from the plain to high altitudes or vice versa, some show almost complete modifications the first year, whereas others only show the beginning of transformation after 10 years. Therefore, all the degrees of plasticity are possible, depending on the species considered” (Bonnier, 1895).

By 1918, Fisher had introduced a method that allowed for the separation of different causes of variability: “It is therefore desirable in analyzing the causes of variability to deal with the square of the standard deviation as the measure of variability. We shall term this quantity the Variance of the normal population to which it refers, and we may now ascribe to the constituent causes fractions or percentages of the total variance which they together produce” (Fisher, 1918). At the time he considered that the variation due to environment was nihil (probably less than five percent) and that most of the variation instead was due to ancestry, Mendelian segregation and dominance. Although later he did reconsider the environment as a possible source of variation and with it the relationship between environmental and heritable variation when he first presented the “analysis of variance” table (Fisher and Mackenzie, 1923; Tabery, 2008), the effect of the environment really created a potential complication for assessing the relative importance of heredity and so it was to be considered and then either dismissed or eliminated or at least minimized by experimental design (Tabery, 2008; Strandberg, 2009). Not for Lancelot Hogben, however, who further developed his thoughts on the relationship between differences of genetic constitution and the external environment in the process of development. He thus recognized three different sources of variability: genetic, environmental, and that which “arises from the combination of a particular hereditary constitution with a particular kind of environment,” or Genotype × Environment interaction (Hogben, 1932; Tabery, 2008).

### Canalization

Meanwhile, in the Soviet Union, the concept of the reaction norm was further developed in the 1920s, such as resulting from the work of Dobzhansky on the “abnormal abdomen” mutation of Drosophila *funebris* (Sarkar, 1999). Much in line with Johannsen, he held that it was the entire reaction norm that was inherited and that mutation resulted in a change in this norm of reaction (Nicoglou, 2014). Subsequently, Schmalhausen (1949; originally published in Russian in 1938) clearly recognized the influence of the environment on the evolution of the reaction norms: different environments will expose different portions of the reaction norm that will be subjected to natural selection, whereas the portions not exposed, or no longer exposed when the environment changes, will be subjected to drift. Changes in the environment eventually result in adaptive modifications that will again “stabilize” into new adaptive phenotypic response curves. The reactivity of the reaction norms, stabilized by means of processes of autoregulation through underlying reactions, would thus be buffered or “canalized” into a more specific optimal norm (Schmalhausen, 1949; Pigliucci, 2001). This idea is similar to that proposed (independently) by Waddington a few years later (1942): “The main thesis is that developmental reactions, *as they occur in organisms submitted to natural selection*, are in general canalized. That is to say, they are adjusted as to bring about one definite end-result regardless of minor variations in conditions during the course of the reaction. (…). The canalization, or perhaps it would be better to call it the buffering, of the genotype is evidenced most clearly by constancy of the wild type.” The constancy of the wild type was recognized earlier by Darwin (1869) when he wrote observing a “much greater variability, as well as the greater frequency of monstrosities, under domestication or cultivation, than under nature.”

Since canalization thus reduces the phenotypic *expression* of variation, it can actually result in the undetected accumulation of selectively neutral underlying genetic variation and mutation accumulation, a concept that is extensively discussed by Schlichting (2008). In other words, the genotype “absorbs” a certain amount of its own variation such as that resulting from new mutations (“genetic canalization”) or that resulting from environmental perturbations (“environmental canalization”; Waddington, 1942; Pigliucci, 2001).

### Genetic Homeostasis

Lerner (1954) coined this ability of a Mendelian population of organisms to equilibrate its genetic composition and to resist sudden changes “genetic homeostasis”, as grounded in the concept of physiological homeostasis proposed earlier by Cannon (1932) (Hall, 2005). Thus, canalization of a character can be equated with homeostasis of that character. In effect, “[b]y insensible gradations this *functional homeostasis* merges with physiological reactions which result in *developmental homeostasis.* (…) A given repertory of functional and developmental homeostatic mechanisms is, of course, determined by the norm of reaction of each genotype” (Dobzhansky and Levene, 1955). And, similar to physiological homeostasis, straying away from the limited variety of possible reaction norms established in evolution under the control of natural selection would result in death (Dobzhansky and Levene, 1955). Although Lerner’s genetic homeostasis was described for Mendelian populations and not for individuals, he argued that it was brought about by the same mechanisms as those which underlie the other forms of homeostasis (Dobzhansky and Levene, 1955). It was implied that Darwinian fitness, resulting from homeostatic adjustment through self-regulation to environmental or genetic disturbances, was manifested by true heterosis or hybrid vigor (Woolf and Markow, 2003). And hybrid vigor, in turn, was considered to be a consequence of heterozygosity, as first proposed independently by Shull and East in 1908, and after by Dobzhansky in 1950. Dobzhansky proposed that it was particularly *coadapted* heterozygosity that was a component of Darwinian fitness, referring to polygene complexes which have become mutually adapted by natural selection in the course of evolution; however, some years later he concluded that heterozygosity may produce higher fitness even without prior coadaptation (Woolf and Markow, 2003). Lerner (1954) also emphasized the heterozygote buffering advantage associated with coadapted polygenic systems resulting from evolutionary history, especially in natural populations, although he also indicated that heterozygosity at a single locus (or coadapted homozygosity in self-fertilizing plants) and epistasis may play a role in determining adaptation (Woolf and Markow, 2003). In addition, he held that no population can afford to maintain too many heterotic loci or blocks simultaneously (Lerner, 1961).

### Phenotypic Plasticity vs Canalization

According to Lerner (1954), the superior buffering ability of heterozygotes at complex multigenic systems would serve two important functions: it would allow for individuals with combinations of phenotypic properties that are expressed near the optimum (canalization), while at the same time it would result in genetic variation, although “hidden” in the phenotypes, and potential plasticity (Woolf and Markow, 2003; Hall, 2005). As Dobzhansky and Levene (1955) note, homeostasis does not prevent the development from switching from one of the historically established paths to other established paths, as long as they remain within the canalized norm. The ability of the organism to follow any of these paths (or to change paths) is, in fact, highly adaptive. This emphasizes the complementary relationship between the processes of canalization and plasticity. Indeed, as given by Waddington (1953) and Dobzhansky and Levene (1955), homeostasis does not imply a stationary state but a dynamic (plastic) stability (canalization); “homeostasis is brought about by changes in some processes which result in stability of other processes.” And following Cannon (1932): “Constancy is in itself evidence that agencies are acting, or ready to act, to maintain this constancy.” Schmalhausen (1949) considered that those animals that are best in responding adaptively to changes in the environment (i.e., those with highest plasticity) while simultaneously best withstood environmental perturbations (i.e., those with highest canalization) would be favored by natural selection (Willmore et al., 2007). Also Bradshaw (1965), in a key contribution to the field, emphasized the adaptive value and evolutionary significance of plasticity, in particular in plants since they are not able, as animals are, to evade adverse conditions: plasticity of certain characters may lead to homeostasis (canalization) of others (Bradshaw, 1965). An example of a plastic mechanism in animals that results in overall robustness (phenotypic stability) is protein turnover, which is responsive to various physiological and developmental scenarios, and provides the flux that is necessary for metabolic regulation and adaptation. Because it is involved in maintenance of homeothermy, reproduction, development, the repair of damaged tissue, maintenance of the immune system, combating infection, and the nutritional/physiological status, a high turnover rate may improve robustness by improving the ability of an animal to adapt to new dietary and physiological conditions (Baldwin et al., 1980; Rauw, 2012). Also plasticity in the functioning of the hypothalamic–pituitary–adrenal axis, which is the most important stress-responsive neuroendocrine system and shows large differences across species, breeds and individuals, has been found to improve robustness through its effects on metabolism, the immune system, inflammatory processes and brain function (Mormède et al., 2011).

Bradshaw (1965) proposed that plasticity of a character can be (a) specific to that character, (b) specific in relation to particular environmental influences, (c) specific in direction, (d) under genetic control, and (e) radically altered by genetic selection. According to Via (1993) and De Jong (1995), “plasticity can be produced either by environment-specific gene expression or by allelic effects that vary across environments.”

## Reaction Norms Analysis in Livestock Production

**FIGURE 1:**
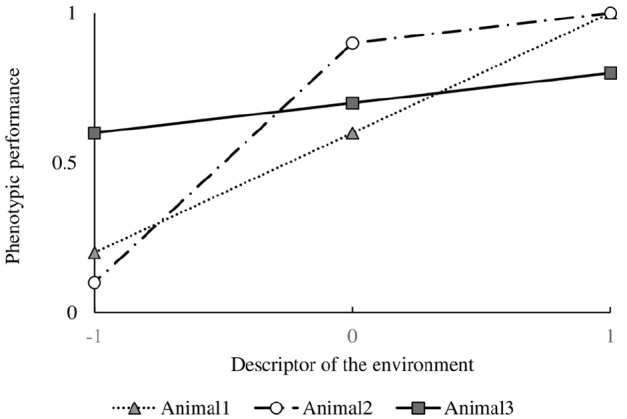
**Phenotypic performance of three different (imaginary) genotypes as a function of an environmental gradient at values –1 (an unfavorable environment), 0 (a “neutral” environment) and 1 (a favorable environment)**.

De Jong (1995) defined the reaction norm as the total pattern of expression of a character along a continuous gradient, and plasticity as the difference in character value between environments, i.e., the first derivative of the function in that environment. When the environment cannot be described along a continuous gradient, than it will be mandatory to describe the phenotypic expression as a series of character states, i.e., values as points on the curve. However, when the environment can be described by a continuous variable, it is possible to describe a character by a function (the reaction norm) and use the function values, coefficients and derivatives for traits (De Jong, 1995). Although reaction norms are mostly described as linear relationships, they can take any shape. Figure 1 presents phenotypic character states of three different (imaginary) genotypes as a function of an environmental gradient at values –1 (an unfavorable environment), 0 (a “neutral” environment) and 1 (a favorable environment). Animal 1 shows a steady increase in phenotypic performance when the environment improves. Animal 2 increases its phenotypic performance slightly when the environment becomes more favorable, however, it is particularly negatively affected when the environment becomes more challenging. Animal 3, as animal 1, shows a steady increase in phenotypic performance when the environment improves, but at a slower rate.

Reaction norms analysis in animal breeding involves quantification of resilience of phenotypic values of production performance expressed by a genotype or by various genotypes across a gradient of a descriptor of the environment (Knap, 2005). Intuitively it holds that an abundant environment will result in a better production performance whereas a restricted environment will depress production. It is proposed that an animal (a genotype) that is best at maintaining its production across this gradient is more robust (i.e., less sensitive) because it has a greater ability to adapt to environmental fluctuations. It is clear from the description that this method is not much more specific than the trait it aims to measure, but it does visualize what robustness represents: a combined production ability (y-axis, the level) and environmental adaptability (x-axis, the slope) trait that can be described in different ways depending on how the variables along the axes are quantified. For example, along the x-axis, environmental factors affecting animal production can be thought to include disease exposure, social stress, stocking density, temperature, nutrient quality, feeding regime, etc. In addition to the ability to maintain production performance, the animal in question will need to be healthy with a sufficient welfare, as it can only be considered robust when its production is qualified as “unproblematic.” In order to include this last part in the analysis, a multi-dimensional representation could be imagined, not only including production traits measured across a gradient of a descriptor of the environment, but also health and welfare traits measured across a gradient of the environment or of the production response.

In plants, a particular individual genotype can be represented by identical clones, however, in animal breeding, the reaction norm of an individual “genotype” (often the sire) can be approximated by its offspring which is spread across a wide environmental range, usually through AI (Knap, 2009). The following three sections give an overview of the use of reaction norms analyses today in dairy cattle, beef cattle, and pigs. The aim of these sections is to review how x- and y-axis traits are formulated in these different livestock species, and to indicate some of the results that followed from the analyses.

### Reaction Norms in Dairy Cattle

The reaction norms method has been mostly applied to dairy cattle, which can count on large numbers of daughters for each sire that are producing at a wide variety of herd environments at which a wide variety of characteristics are recorded. This wide range of available production characteristics facilitates investigation of a descriptor of the environment as a continuous variable instead of being limited to describing the environment as discrete classes, i.e., as a series of character states. Zwald et al. (2001), Fikse et al. (2003), and Calus and Veerkamp (2003) describe several continuous climate and herd management characteristics that can be used as descriptors of the environment, such as “mean peak yield” and “persistency” as an overall measure of the quality and intensity of the management system, “days to peak yield” reflecting differences in dry cow management and health and nutrition programs, “herd size” as an indirect measure of differences in facilities and treatment of cattle, “day of calving” as a variable that could separate rotational grazing herds with seasonal calving from other types of herds that feature year-round calving, “percentage of animals with completed lactations” as a measure to explain differences in culling strategies between farms, “fat:protein ratio” as a measure of the feeding system, “body condition score” as a measure of the ability of management to tune the feed intake to the energy requirements of the animal, and a temperature and humidity indicator as a measure of the heat stress on cows. As Calus and Veerkamp (2003) indicate: “Potentially a large number of environmental parameters could be defined, but parameters used (…) were chosen because they: (1) reflect management and environment, (2) are obtainable from the available data, (3) are continuous rather than categorical (…), and (4) are not too strongly correlated with each other.”

Strandberg et al. (2000) used the herd-year effect as a general measure of a complex of environmental values to which they linearly regressed 305-d protein yield and days open to estimate breeding values in Nordic dairy cattle (Finnish Ayrshire, Norwegian Dairy Cattle, and the Swedish Red and White Breed). Crossing of reaction norms indicated reranking in the presence of genotype × environment interactions for both traits. Calus et al. (2002) performed a random linear regression of 305-d heifer protein production on herd-year-season in Dutch Holstein Friesian dairy cattle. The level of the reaction norm had such a great impact that the slope had very little influence on the total breeding value, and no genotype × environment interaction was observed. They suggested that another more environmental-specific parameter or defining another scale for the environmental parameter might contribute to increase the influence of the slope. In addition, they suggest that non-linear reaction norms might explain sire variance better. Ravagnolo and Misztal (2002) estimated the genetic component in heat tolerance for non-return rate in Holstein cows using an animal linear model augmented by a random regression on a temperature-humidity index. They observed a negative, unfavorable genetic correlation between merit for milk yield and non-return rate at 90 days after first insemination but indicated that simultaneous selection for improving both traits is feasible. Kolmodin et al. (2002) regressed first lactation 305-d protein production and days open on the herd-year average in Nordic dairy cattle (Danish Red Dairy Breed, Finnish Ayrshire, Norwegian Dairy Cattle, and the Swedish Red and White Breed). They evaluated three different reaction norm models: (1) a random regression on an environmental variable, (2) a regression model including the level and the slope of the reaction norm of the sire, and (3) an extension of model (2) to include a set of regressions on a second environmental variable. The models were similar in both the level and the slope. Results showed that the genetic parameters changed over environments, and that a significant variation for the slope of the norm resulted in little reranking of sires, except between extreme environments. Fikse et al. (2003) regressed 305-d milk yield on fifteen environmental parameters in Guernsey-sired cows (from Australia, Canada, United States, and the Republic of South Africa). Nine parameters had a significant effect and results indicated that reranking of animals may occur in extreme environments. Calus and Veerkamp (2003) estimated breeding values for milk, fat, and protein yield and percentage, of daughters by applying a random regression on various values of environmental parameters for each sire in Dutch dairy cattle (mostly Holstein-Friesian and Meuse-Rhine-Yssel). Twelve of fourteen environmental parameters gave significant reaction norms, but reranking hardly occurred across environments. Hayes et al. (2003) investigated the magnitude of genotype × environment interactions of milk, protein, and fat yield from a random regression on four different environmental descriptors in Australian Holstein-Friesian dairy cattle. Interactions were observed for average herd protein yield and temperature humidity index. Bryant et al. (2006) investigated the environmental sensitivity of Holstein Friesian and Jersey dairy cattle and their crosses for 2-year milk, fat and protein yields in relation to the range of herd milksolid yields (as a proxy for feeding level) in New Zealand using first and second degree polynomial regression functions. Their results indicated that Holstein Friesians originating from overseas (mostly from North America), exhibited higher levels of production (level) but also higher environmental sensitivity (slope) than Holstein Friesians from New Zealand and Jerseys. The overseas Holstein Friesians, which are selected in an environment where high levels of concentrate are offered and high levels of production are achieved, improved their ranking in a high production level environment, whereas New Zealand Jerseys, which are selected in pasture-based, low production level environments with high levels of environmental heterogeneity due to the variable nature of pasture supply, improved their ranking in a low production level, grassland-type environment. Haile-Mariam et al. (2008) regressed not only milk production traits (milk, fat, and protein yield and percentage) but also fertility traits (calving interval, calving to first service interval, 25-d non-return rate at first service, and pregnancy rate) and survival to the next lactation on the environmental descriptors “level of herd milk production,” temperature-humidity index, and herd size in Australian Holstein-Friesian dairy cattle. There was no evidence for the presence of a large genotype × environment interaction that resulted in economically significant reranking of bulls. Shariati et al. (2007), fitting a reaction norms model to first test-day records for first lactation milk, protein, and fat of Danish Holstein cows, reported the presence of genotype × environment interaction, but with a small effect on reranking of candidates for selection. Streit et al. (2012) applied reaction norm random regression sire models to corrected test day records for milk, protein, and fat yield and somatic cell score as a function of herd test day solutions as environmental descriptors in German Holstein dairy cattle. Results indicated the presence of minor genotype × environment interactions which did not result in reranking of sires.

### Reaction Norms in Beef Cattle

Corrêa et al. (2010) evaluated differences in sire genetic values by a reaction norms hierarchical model for post weaning gain in response to estimates of contemporary group effects in Brazilian Devon cattle. They reported the existence of genotype × environment interaction. Most reranking of sires happened in restrictive environments, indicating that importing genetic material should be carefully assessed when the selection conditions of the animals in the exporting countries are greatly superior to local production environmental conditions. Pégolo et al. (2009) assessed genotype × environment interaction for 450-day adjusted weight and body weight gain in Brazilian Nelore cattle using a random regression reaction norms model on heard-year and herd-year-season-management groups, and heard-year-season-management group solution estimates. The models generated consistent parameter estimates. Important genotype × environment interactions were found with low genetic correlations among extreme environments, indicating a significant reranking of sires in different environments. Mattar et al. (2011) investigated the presence of genotype × environment interactions for long-yearling weights of Brazilian Canchim cattle using reaction norms of the trait as a response to a “contemporary group” effect that combined year and season of birth, sex, genetic group of dam, herd at weaning and long-yearling, and feeding regimen from birth to weaning and from weaning to long-yearling. Their results showed that all animals increased their performance with the environmental improvement, that there was some reordering of genotype ranks, and that there existed variability in phenotypic plasticity. Cardoso and Tempelman (2012) investigated alternative linear reaction norms models for post-weaning body weight gain to a “contemporary group” effect of herd-year-season-sex-management subclasses in Brazilian Angus cattle. They observed genotype × environment interactions and possible reranking, and furthermore concluded that environmental sensitivity of imported North American Angus bulls was significantly larger than that of local Brazilian Angus sires which tended to be more robust to environmental changes. Santana et al. (2013) determined the presence of genotype × environment interaction for birth weight, weaning weight, postweaning weight gain and yearling scrotal circumference in Brazilian composite beef cattle from reaction norms taking the environmental covariate of the reaction norms (the contemporary group) as the environmental descriptor. A genotype × environment interaction was observed and reranking of animals and it was concluded that it can be important to include phenotypic plasticity in the breeding goal.

### Reaction Norms in Pigs

Reaction norms in pig production are scarcely described. Knap and Su (2008) estimated linear reaction norms of total litter size at birth as a function of routine herd-year-season effects in two PIC lines of pigs and their cross. Daughters of sires were spread over North and Latin America, Europe, Asia and Australia, providing for a wide range of environmental effects of a climatic, nutritious, management-related and infectious nature. Environmental sensitivity showed a progressively lower genetic component with increasing data volume, and progressively less frequent reranking of genotypes across the environmental range. Consequently it was recognized that reaction norms analysis is indeed a demanding process, requiring large data volume and a wide environmental range in order to produce meaningful results (Knap and Su, 2008).

### Reaction Norms for Behavior and Welfare

So far, a behavioral reaction norm as suggested here previously has not been applied in livestock production, however, Sih et al. (2004) proposed that behavior can be included in phenotypic plasticity and reaction norms models. Similar, Dingemanse et al. (2009) describe that animal responsiveness (behavior) can be described as a function of environmental variation (context), and that this can be considered a complementary aspect of the individual phenotype. Examples given are the relationships between parental provisioning rate and offspring begging intensity, between dispersal behavior and wind velocity, or between anti-predator behavior and predation risk (Dingemanse et al., 2009). In addition, animal personality is suggested to express itself as a coping strategy that is consistent across contexts (Koolhaas et al., 1999); Sih et al. (2004) refer to such suites of correlated behaviors in an individual as “behavioral types,” which show consistency in behavior across multiple situations. This behavioral consistency may be represented by the individual behavioral response as a function of a stimulus that can vary across a gradient, as an index of its behavioral stability (Sih et al., 2004; Dingemanse et al., 2009). Personality does not imply that each individual is necessarily completely consistent in behavior, such that variation in plasticity may be observed between individuals and populations (Dingemanse et al., 2009). Coping styles are important in livestock production as they form general adaptive response patterns that have genetically evolved in reaction to everyday challenges and are thus closely related to individual adaptive capacity and vulnerability to stress-related disease (Koolhaas et al., 1999). Dingemanse et al. (2012) used the reaction norms approach to estimate the quantitative genetics parameters of the exploration behavior of an open-field of over 1000 offspring of two populations of wild-caught three-spined stickleback fish. They found heritable variation and population differences in both the average level of exploration and behavioral plasticity.

Examples in livestock production of environmental gradients can be thought to include group size and composition, temperature, photoperiod, environmental enrichment, but might also include production parameters such as growth rate or milk production. Smiseth et al. (2008) described behavioral reaction norms to investigate parent-offspring conflict and co-adaptation. They indicate that behavioral interactions can include other questions where the expression of traits depends upon the behavior of other individuals, “encompassing the whole field of animal communication,” such as aggression related to competition for resources. A similar analysis may be applicable to social interactions in livestock production systems.

### Selection for Increased Production, Against Environmental Sensitivity

The breeding value as estimated from reaction norms analysis is built up of two parts: the environment-independent part (the level), and the environment-dependent part (the slope; Calus et al., 2002). Thus, the ideal reaction norm in animal production has a high level and a flat slope (Strandberg et al., 2000). According to De Jong (1995), the level and the slope are genetically correlated; however, this does not necessarily mean that separate genes for plasticity and trait mean exist.

Su et al. (2006) indicate that in reaction norms analysis a linear relationship between the phenotypic expression of a given genotype and the covariate representing a particular environmental effect is assumed, which is approximated by using the mean phenotypic performance in the appropriate environment, without the need to know the actual covariate. However, the variance among phenotypic means of production environments includes a genetic component, resulting in overestimation of the variation of environmental values, even in a random mating population. In addition, computer simulation indicated that it results in an underestimation of variance components associated with the slope, and an overestimation of the variance components associated with the level. Instead, they suggest a more satisfactory alternative by inferring environmental values simultaneously with the other parameters in the model using a Bayesian Markov Chain Monte Carlo approach, which was shown to lead to estimates of parameters with no detectable bias and with smaller mean squared errors. To account for a scale effect on residual variances in reaction norms models such that larger environmental effects are associated with larger residual variances, Cardoso and Tempelman (2012) proposed two alternative extensions to the model to allow for heteroskedastic residuals: an exponential function and a best fitting environmental classification model; the latter seemed to provide a better fit than the exponential function.

Lillehammer et al. (2007, 2009) described a different approach by investigating not the effects of genotypes but the effects of single genes in response to environmental variation using quantitative trait loci (Lillehammer et al., 2007) and single nucleotide polymorphisms (Lillehammer et al., 2009). This is important since QTLs and SNPs with an environmental interaction can be hard to detect even though they have a large average effect. In the SNP analysis they report a genetic correlation between general production and environmental sensitivity from 0.55 to 0.88, indicating that most genes should affect the level and the slope in the same direction. This supports earlier work by Kolmodin et al. (2002) who observed that animals with genetically high production tended to be more sensitive to changes in the production and fertility environment, and by Kolmodin et al. (2003), who studied the effect on environmental sensitivity (the slope) of selection for high phenotypic value (the level) in combination with a continuously improving environment in a simulation study. They detected a significant selection response, suggesting that environmental sensitivity will increase with selection for high phenotypic values. These observations were also supported by later work, for example by Knap and Su (2008), who indicated that the very precisely estimated correlation between the intercept and the slope was extremely high: “Hence, irrespective of genetic effects, the performance of sows with a high reproductive capacity is practically always highly sensitive to environmental disturbance. [The same pattern applies to] the genetic level; [it is clear] that for litter size, the performance of high-potential genotypes (and of high-capacity sows) will likely come down strongly when environmental conditions become unfavourable.” However, because of the low heritability of the slopes, environmental sensitivity would be increasing at a slow rate.

The negative correlation between high levels of production and increased environmental sensitivity can result from resource allocation patterns described by Beilharz et al. (1993). Resource demanding physiological processes show trade-offs resulting from limits in the resource availability, food intake and digestive capacity and/or limiting resource allocation patterns which typically result in a genotype × environment interaction. Animals that are genetically driven to produce at high levels may need to reallocate resources away from other process, leaving the animal lacking in ability to respond to other demands, such as coping with disease and stress. This will consequently result in an animal that is more sensitive to environmental fluctuations (Rauw, 2009). Indeed, Friggens and Van der Waaij (2009) indicate the single-trait limitation of the reaction norms approach and developed resource allocation models, based on the model of Van der Waaij (2004), providing a framework for a multi-trait definition of robustness. This model explicitly examines the partition of resources between different life functions and provides a framework for exploring trade-offs. The equations allow for relating total fitness to environmental variation and resource availability, defining plasticity in terms of more than one trait. This is more biologically meaningful since adaptation to environmental change is essentially a process that results from a combination of physiological mechanisms (Friggens and Newbold, 2007). However, as reviewed by Friggens et al. (2013), the challenge of linking prediction of nutrient partitioning to its consequences on health, reproduction, and longevity is only recently being addressed, and so far the models developed, for the most part, remain research models that need to be further developed to be applied in the field.

As Kolmodin et al. (2003) notes, high sensitivity may be beneficial when the environment is highly controllable and predictable, since the benefit from improvements of, for example, management and feeding would be substantial, while the risk of environmental deterioration, causing drastic reductions in levels of production, would be relatively low. However, since populations of animals with high production potential will be more dependent on highly controlled environments this may be of ethical concern. Lillehammer et al. (2009) indicate that their results show that a small fraction of the genes affect only production (the level) or only environmental sensitivity (the slope). In addition, even a category of possible selection gene candidates was found that affects production and environmental sensitivity in opposite directions. Such genes would facilitate selection for increased production and robustness at the same time.

## Direct Inclusion of Robustness Traits in the Breeding Objective

The second option for breeding for animal robustness is the direct approach, which encompasses the inclusion of directly measurable robustness traits in the breeding objective and in the selection index. These robustness traits can include the same physiological, immunological and reproduction traits that are affected as a result of selection for high production efficiency (Rauw et al., 1998). They are often referred to as “functional traits,” i.e., traits that are closely related to biological functional ability or fitness, such as longevity, health and fertility. Although these traits are important to all livestock animals, the term is mostly used in dairy cattle production, where they can include structural soundness, udder and teat conformation, frame score, disposition/temperament, body condition score, fertility, calving ease and mothering ability, and adaptability to the environment (Peck, 2006; Egger-Danner et al., 2015). Similar fitness traits related to longevity, health and fertility are described for other livestock species. The Nordic countries (Sweden, Norway, Denmark) in particular have broadened breeding goals to also include fertility and health, which became possible since these countries implemented well-established, national recording systems for health data (Herringstad et al., 2000). Since the mid-1990s also several European and North-American breeding organizations have included fertility and health in their breeding objectives (Oltenacu and Broom, 2010). The International Committee for Animal Recording (ICAR) promotes since 1951 the development and improvement of activities of performance recording and the evaluation of dairy cattle and its Functional Traits Workgroup is in particular involved with recommendations regarding functional traits in dairy cattle. Heritabilities of functional traits and feasibility of inclusion of these traits in the breeding objective has been described in a number of works and several reviews (e.g., Groen et al., 1997; Essl, 1998; Herringstad et al., 2000; Lawrence et al., 2004; Egger-Danner et al., 2015). According to Knap (2009), genetic improvement of robustness traits can improve profitability of production at a similar rate as by improvement of a conventional production trait. In spite of antagonisms between robustness and production performance, a positive genetic trend in both traits can be achieved at the same time when robustness traits are properly included in the breeding goal and selection criteria (Knap, 2009).

In addition, several authors discussed the feasibility of including behavioral traits that are related to animal welfare in the selection criterion. These traits will improve animal welfare and can be expected to lead to improvements in mortality, disease resistance, efficiency, longevity, reproductive performance and carcass wastage as a correlated effect (Turner, 2011). For example, Jones and Hocking (1999) extensively reviewed the feasibility of using selective breeding to improve welfare, describing results of selective breeding studies in which fear, adrenocortical stress responses, social motivation, feather pecking, and growth rate were manipulated in quail and chickens. Star et al. (2008) described including, besides immunological and physiological traits, also behavioral traits in laying hens. Rydhmer and Lundeheim (2008) proposed to include improved piglet survival, stronger legs, a better constitution, improved disease resistance, less aggressive behavior, reduced fear of humans and a great appetite in the breeding programs of pigs. D’Eath et al. (2010) discussed the possibilities of selection for farm animal behavior in livestock species in general, indicating that in many cases, estimated heritabilities are of comparable magnitude to traits already included in the breeding program (0.1 to 0.4) which suggests that selection for behavior would result in a positive selection response. Turner (2011) explored the genetic contribution to harmful social behavior traits using as examples regrouping and poor maternal care in pigs, and oral manipulation of penmates in pigs and laying hens, and concluded that for most traits, improvements in harmful behavior can be made by careful breed choices and selective breeding. Dawkins and Layton (2012) describe the feasibility of breeding for better welfare in broiler chickens, noting that “Broiler chicken welfare is most likely to be improved in practice if animal welfare traits such as good walking ability, good feathering and healthy legs and feet are seen as compatible, rather than in conflict, with other goals such as commercial production.” Canario et al. (2013) reviewed the feasibility of including behavioral traits in the selection criteria of cattle, pigs, poultry and fish. They note that animal behavior is a welfare indicator since it relates both to the existence of stressors and to the animal’s ability for behavioral adaptation to physical and social environmental stressors. Mormède et al. (2011) proposed to select animals for a higher activity of the stress-related hypothalamic–pituitary–adrenal axis (which releases cortisol or corticosterone) to improve animal robustness and welfare. And finally, Oliveira et al. (2010) proposed assessment of play behavior as a new and promising potential indicator of animal welfare. According to Allen and Bekoff (2005), there are evident emotions associated with play—joy and happiness—that drive animals into it. Indeed, animal play only if they are healthy, safe, well-fed and in a relaxed state, but not if they are under a stressful condition (Burghardt, 2005). According to Held and Špinka (2011), play may signal both the absence of bad welfare and the presence of good welfare, however, it does not consistently reflect favorable environmental conditions. Rauw (2013) investigated the consistency of a behavioral play marker in piglets and proposed investigating the feasibility of using play markers in the selection criterion of livestock species.

The challenge to including behavioral traits in the selection criteria is to define quantifiable traits or proxy measures thereof that can be recorded cost-effectively and reliably on the large number of animals that are necessary for a breeding program (D’Eath et al., 2010; Turner, 2011). In addition, which trait(s) to select for in order to truly improve animal welfare is complicated by the many different conceptions and definitions of animal welfare proposed, defined in terms of, e.g., animal function, the balance of enjoyment or pleasure vs. suffering or pain, preference satisfaction, or natural living (Duncan and Fraser, 1997; Lassen et al., 2006). As Turner (2011) notes, it may be difficult to identify behavior in the recipients vs. the donors (for example of aggression), and it may be challenging to attribute an accurate economic value to behavioral traits. In addition, D’Eath et al. (2010) warn for selecting animals that do no longer show outside signs of negative welfare, but still experience the negative feelings associated with the unwanted behavior, for example in the case of docile animals that are too frightened to move. It may thus be necessary to first further investigate the cognitive processes and emotional experiences underlying the phenotypes (Turner, 2011).

Finally, in addition to production traits, functional traits and behavioral traits, Olesen et al. (2000) discussed the need to define animal breeding goals as an integrated part of sustainable production systems, i.e., based on a holistic, long-term perspective. They stress that higher productivity should not only be balanced with (short-term) improved health, fertility, and feed intake capacity, but also with (long-term) important non-market values of animal traits, such as ethical values of improved animal welfare and possibly also with natural capital and ecosystem services (depletion of fossil energy, degradation of the atmosphere) and social issues. Also Kanis et al. (2005) proposed including “societally important” traits, such as product safety, welfare, and environmental impact, which do not have a clear economic value. They present a retrospective selection-index method to obtain the proper weights for those traits. Olesen et al. (2000) emphasize that animal breeding practices must become part of the pluri- and interdisciplinary, philosophical and ethical debate. Code-EFABAR also follows the principles of sustainable breeding in their Code of Good Practice; the general definition of sustainable farm animal breeding is defined as: “the extent to which animal breeding and reproduction, as managed by professional organizations, contribute to maintenance and good care of animal genetic resources for future generations” (Gamborg and Sandøe, 2005; Code-EFABAR, 2006).

## Ethical Considerations

Artificial selection was already described by Mago from Carthage in his work “Treatise on Agriculture” several centuries BC in which he recommended choosing oxes that were “young, stocky, sturdy of limb with long horns, darkish and healthy, with a wide and wrinkled forehead, hairy ears and black eyes and chops, the nostrils well-opened and turned back, the neck long and muscular, and dewlap full and descending to the knees, the chest well-developed, broad shoulders, the belly big like that of a cow in calf, the flanks long, the loins broad, the back straight and flat or a little depressed in the middle, the buttocks rounded, the legs thick and straight, the hooves large, the tail long and hairy and the hair on the body thick and short, red-brown in color and very soft to the touch” (Koster, 2015). Selective breeding has been responsible for the domestication of 14 animal species and about 100 plants yielding valuable domesticates (Diamond, 2002). Before the 1940s, breeding objectives were mostly visual with the expectation that form determines performance (Darlow, 1958). Subsequently, breeding industries evolved toward objectives involving performance, such as rapid growth and high milk yield (Harris and Newman, 1994). Breeding value estimation was limited to the data that was available for evaluation. This first included single traits, until models were developed for combining several traits into a selection index by Hazel and Lush (1943), and methods were developed such as those for the estimation of variances and covariances for unbalanced animal data by Henderson (1953) (Philipsson et al., 1994). In dairy cattle, as reviewed by VanRaden (2004), the national index of Swedish dairy cattle included 12 traits in the selection index as early as 1975, including milk production, growth rate, female fertility, stillbirths, ease of milking, temperament, and six conformation traits (Philipsson et al., 1994), but the USDA introduced its first net merit index in 1994, which combined productive life, and somatic cell score with yield traits (VanRaden, 2004). The USDA selection index subsequently included conformation traits in 2000 and cow fertility and calving ease in 2003.

Only recently is selection for production traits under scrutiny for the *consequential* undesirable side effects that this may produce affecting animal welfare (Rollin, 1986), thus leading the British Farm Animal Welfare Council to recommend that new and existing breeding technologies and breeding programs should be evaluated for welfare and ethical issues that may arise as a result (FAWC, 2004). Broadening the breeding objective and including more traits in the selection criteria may alleviate and possibly even prevent such negative side effects, with as the only negative consequence a reduced selection response of production traits. However, genetic modification may also result in an *intrinsic* ethical concern when breeding affects animal integrity. Rollin (1986, 1995) used the Aristotelian concept of the *telos* of an animal to describe animal nature, i.e., the differences “rooted in biological, genetically based, empirically ascertainable, environmentally expressed “blueprints”” giving rise to “the pigness of a pig, the dogness of a dog.” Bovenkerk et al. (2002) write: “It implies that the animal is intact or whole, which is an attribute of the animal itself, not just some value we have placed on it.” Any artificial genetic modification may be seen as changing the *telos*, however, D’Eath et al. (2010) suggest that animal behavior is much more easily considered to be part of the animal’s nature than any other production trait. As to the question of changing the telos by means of changing the genetic make-up, Rollin (1986) writes: “[O]ne cannot argue that because it is wrong to violate the various aspects of a certain animal’s *telos* given the *telos*, it is therefore wrong to change the *telos*. This is true only if the change in the *telos* is likely to engender more unhappiness in the animals, given the environment in which they live, than would have accrued to them before” (Rollin, 1986). Indeed, Rollin believes that there is no moral problem if welfare could be *improved* by changing animal natures, even altering animals such that they can be made happier in questionable environments (Rollin, 1995; Bovenkerk et al., 2002). For example, animals bred to have fewer desires or animals with a reduced sentience will be more easily satisfied and consequently have a higher welfare than the population before such selection (D’Eath et al., 2010). In the same way, blind chickens do not show feather pecking or cannibalism, therefore, blind hens may not suffer (Sandøe et al., 1999). Strains that are improved to disguise welfare threatening conditions may discourage the development of higher standards of environmental provisioning (MacArthur Clark et al., 2006). As a consequence, in extreme cases, genetic modification of animals into senseless, emotionless machines that have no desires could be considered a solution to the animal welfare problem.

However, intuitively, a large amount of the human population believes that genetic modification of animals is troubling and morally problematic; as such the public opinion can be expected to influence breeding decisions made by producers that would eventually prevent producing animal machines (Rollin, 1998; Thompson, 2010). As Bovenkerk et al. (2002) note, animal integrity is an intuitive concept, and because it lacks objectivity it is therefore not of practical use since that would entail objective criteria to measure it. However, not different from ethical considerations in humans, the concept of integrity can be used in the ethical discussion on livestock breeding, and in the same way that concepts of human rights based on integrity are formulated into laws, from discussions regarding the ethics of livestock breeding can follow similar agreements and regulations (Bovenkerk et al., 2002). Rauw (2015) suggests that although consumer demand may influence decision making and although consumers may be willing to pay more for products that are produced in more welfare friendly production systems, legislation should really be based on ethics independent of consumer demand and willingness to pay. Similar to the option to buy clothes cheap that are produced unethically versus paying more for clothes that are produced under humane circumstances, we as consumers should not be able to have that option (Rauw, 2015).

The Farm Animal Welfare Council, in its 2012 report, writes: “[In 2004] we were concerned about general trends in breeding, given the commercial pressures on breeders and farmers alike. Today matters are improving: we still have concerns but we are encouraged that many breeding goals now include aspects of animal welfare, e.g., disease resistance.” Conclusion 105 of the report reads: “Farm animal breeding companies should be congratulated for the progress made on breeding goals aimed at improving robustness and health and welfare traits. However, there are still some issues associated with high production levels resulting in poor animal welfare.”

The discussion on animal breeding practices has been initiated and is very alive today. This positive trend is part of the sustainable food production movement that aims at feeding 9.15 billion people not just in the near future but also beyond. However, the discussion is taking place in Europe and North America which are home to the largest livestock breeding companies that hold most of the market share (Gura, 2007). These developed countries are projected to account for only part of the increase in meat consumption, whereas more than half of the increase is projected to be accounted for by developing countries in Asia, Latin America, and Africa, countries that still depend heavily on agriculture for their livelihoods (Borlaug and Dowswell, 2005; Thornton, 2010; Appleby and Fuentesfina, 2015). Although the technology and genetic resources are available, this may be of limited use to local farmers when they are threatened by poverty, governmental regulation and intellectual property rights (Borlaug and Dowswell, 2005). In addition, concern for animal welfare and rights is generally stronger in Europe than in Asia (Phillips et al., 2012) and it remains to be seen if European (breeding) companies will apply their animal welfare standards on a global basis, as suggested by Fraser (2008), or whether this may eventually influence breeding decisions in the future when such standards are not required by international food companies and their customers.

## Synthesis

Since environmental resources (land, water, and energy) are limited, a 70–100% increase in the projected need for food by 2050 must necessarily come from what is called “sustainable intensification.” As Godfray et al. (2010) write: “A threefold challenge now faces the world: Match the rapidly changing demand for food from a larger and more affluent population to its supply; do so in ways that are environmentally and socially sustainable; and ensure that the world’s poorest people are no longer hungry.” Increasing production limits both in crops and in livestock are inevitably part of satisfying the global food demand in the future. A further increase in livestock yields with continued selection will be facilitated by superior selection methods including genome-wide selection, more sophisticated progeny testing and tracking methods, and a greater predictive power of total genetic merit indices that integrate genomic markers with multiple traits (Hume et al., 2011). However, at the same time, animals in populations that have been selected for high production efficiency are found to be more at risk for behavioral, physiological and immunological problems (Rauw et al., 1998). As a result, in the last few decades, breeding practices have become of ethical concern and consideration of the possible effects on animal welfare are called for (e.g., FAWC, 2012).

The farm animal of the future is described as robust, adapted, and healthy (Mormède et al., 2011). Options for breeding for improved robustness include: (1) estimating breeding values for the environmental sensitivity of the genetic potential for production performance through the use of reaction norms analysis, and (2) direct inclusion of measureable robustness traits in the breeding objective and in the selection index (Knap, 2009). Theories on reaction norms analysis have their basis in genotype by environment interactions that have been described since Lamarck and Darwin. Reaction norms describe phenotypic production values as a function of a gradient of a descriptor of the environment (Knap, 2005). They were first applied in plants, whereas application of reaction norms analysis in livestock production (mostly dairy and beef cattle) is of a much more recent origin. Linear reaction norms are built up of two parts: the level and the slope. A generally observed negative correlation between these parameters suggests that improvement in production yield will result in animals that become more sensitive to changes in the production environment (Kolmodin et al., 2002).

Although livestock selection indexes include multiple, mostly yield-related, traits for several decades, direct inclusion of functional, robustness, traits became more seriously applied since the 90s (Oltenacu and Broom, 2010). Of more recent origin is the consideration of inclusion of behavioral traits (Turner, 2011) and even important non-market values of animal traits, such as ethical values or environmental impact (Olesen et al., 2000). Despite an often antagonistic relationship between robustness and production performance, a positive genetic trend in both traits can be achieved when both are properly included in the breeding goal and selection criteria (Knap, 2009).

According to the Farm Animal Welfare Council, farm animal breeding companies may be congratulated for the progress made so far toward breeding more robust and healthy animals. The discussion and efforts on animal breeding practices is very alive today and will remain to be an important part of the sustainable intensification debate in the future.

### Conflict of Interest Statement

The authors declare that the research was conducted in the absence of any commercial or financial relationships that could be construed as a potential conflict of interest.
